# Biogeography and environmental preferences of *Butia yatay* (Mart.) Becc

**DOI:** 10.1002/ece3.10749

**Published:** 2023-11-27

**Authors:** G. Carolina Silva, Viviana Griselda Solís Neffa, Gabriela Zuquim, Henrik Balslev

**Affiliations:** ^1^ Laboratorio de Citogenética y Evolución Vegetal Instituto de Botánica del Nordeste (UNNE‐CONICET) Corrientes Argentina; ^2^ Facultad de Ciencias Exactas y Naturales y Agrimensura Universidad Nacional del Nordeste Corrientes Argentina; ^3^ Department of Biology University of Turku Turku Finland; ^4^ Department of Biology‐Ecoinformatics and Biodiversity University of Aarhus Aarhus C Denmark

**Keywords:** Arecaceae, hindcast, niche modeling, Quaternary, soil characteristics, tropical America savanna

## Abstract

During the Quaternary, Chaco Phytogeographic Domain (Chaco) flora in subtropical South America experienced temperature and humidity fluctuations, primarily driven by wind dynamics, leading to significant shifts in species distribution. The palm *Butia yatay* is endemic to the Chaco and thrives in areas characterized by a warm–rainy climate and mostly restricted to sandy soils. To investigate the current geographic distribution of suitable habitat for *B. yatay* while assessing the significance of soil variables, we employed two distinct algorithms in species distribution modeling (SDM). We also determined whether the distribution of *B. yatay* has changed since the Pleistocene and whether these changes align with previously proposed Pleistocene refugia. In the present SDMs, we considered two separate sets of predictors, one set with bioclimatic variables only and the other set with bioclimatic topographic and soil variables. Additionally, we reconstructed the historical geographic distribution of suitable habitats using bioclimatic data. Our results suggested that the primary determinants of *B. yatay*'s current distribution include precipitation and temperature of the driest month and soil cation exchange capacity. Incorporating soil variables affected the estimated size and range of suitable areas. Projections into the past indicated similar suitable habitat distributions during interglacial periods compared with the present. During the Last Glacial Maximum, climatically suitable habitat may have shifted northward, partially overlapping with previously suggested Pleistocene refugia located between the Paraná and Uruguay Rivers. These findings indicate the main factors driving the distribution and ecology of *B. yatay* and enhance understanding of subtropical flora shifts during the Quaternary. The approach also may prove valuable for other studies within the Chaco.

## Graphical abstract

In this study, we focus on the subtropical palm species, *Butia yatay*, recognized as an endemic flagship taxon within the Chaco Phytogeographical Domain. We determined its current environmental preferences and assess potential shifts in the distribution of *B. yatay* since the Pleistocene. Our results represent a significant contribution to the understanding of past changes in the subtropical flora of South America.

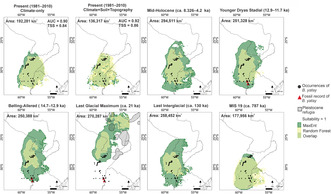

## 1 INTRODUCTION

Tropical America geomorphological processes include hydrological and topographic changes caused by the uplifting of mountains (Hoorn et al., 2010). During the Miocene, the uplift of the Andes and subsidence of the Brazilian craton generated a lowland zone called the Chaco‐Pampa plain (Kruck et al., 2011). This plain constitutes a significant sedimentary basin within central South America, currently modified by river activity (Iriondo, 2010), and the region is included in the Chaco Phytogeographic Domain (Chaco; Cabrera & Willink, 1973). During Quaternary climatic fluctuations, the Chaco‐Pampa plain remained unglaciated. Wind patterns from Atlantic and Pacific anticyclones heavily influenced the climate, subsequently impacting the plain's temperature and humidity (Iriondo, 2010). During ice ages, winds from the south and northwest generated drier and colder conditions. In contrast, in interglacial periods, warm northern winds ushered in increased humidity (Ab'Sáber, 1977; Iriondo & Garcia, 1993). During the dry seasons, pronounced eolian activity has redeposited large masses of silt and sand over the area. In addition, sediments have been transported by fluvial systems such as the Paraguay, Paraná, Uruguay, and Negro Rivers, which formed alluvial fans and terraces as they shifted their courses (Iriondo, 1999; Panario & Gutiérrez, 1999; Popolizio, 2006).

Quaternary geoclimatic changes have influenced species' historical biogeography and population structure (Ab'Sáber, 1977; Iriondo, 1999). In the lowlands, intermittent expansion and contraction has led to species dispersal and subsequent isolation (Baker et al., 2020). These isolated regions, where the biota persisted with reduced distribution and abundance during glacial periods, are called “refugia” (Bennett & Provan, 2008). The Pleistocene refugia hypothesis proposes that glacial cycles promoted forest fragmentation and their replacement by savannas, isolating populations within climatically suitable areas (i.e., refugia; Ab'Sáber, 1977; Haffer, 1969; Prance, 1982; Whitmore & Prance, 1987). The hypothesis also proposes that species that were adapted to wetter conditions migrated to equatorial regions during drier periods and persisted on upland slopes (Ab'Sáber, 1977; Clapperton, 1993). Alternatively, palynological evidence from the Last Glacial Maximum (LGM; ca. 21 ka) indicates that local‐scale changes did not impact entire ecosystems, which challenges the existence of Pleistocene refugia (Bush & de Oliveira, 2006; Colinvaux et al., 2000) and suggests that savanna expansion occurred mainly in the early–middle Holocene (Mayle et al., 2004).

In the context of subtropical lowlands, the Pleistocene refugia hypothesis has received limited attention (Turchetto‐Zolet et al., 2013), and studies have emphasized the herbaceous layer within forests, grasslands, and savannas (Moreno et al., 2018; Speranza et al., 2007). Our focus is on a subtropical palm tree that occurs in lowland areas. Palms (Arecaceae/Palmae) are distributed throughout the tropics and subtropics (Morley, 2000) and originated during the Early Cretaceous. These palms play an important role in biogeographic theory and serve as paleo‐indicators (Baker & Couvreur, 2013; Dransfield et al., 2008). Paleoecological studies have attempted to answer how changes in past climatic conditions influenced the current distribution of palms (da Silva Carvalho et al., 2017; Kissling et al., 2012; Tovaranonte et al., 2015), but climate is not the only driver (Bogotá‐Ángel et al., 2021). Soil quality, topography, hydrology, and other geological conditions also contribute significantly to their current distribution (Eiserhardt et al., 2011; Muscarella et al., 2020). Most of these studies have focused on tropical palms, however, leaving research gaps regarding subtropical palm ecology and biogeography (Bueno et al., 2017; de Lima et al., 2018; Escobar et al., 2021; Trénel et al., 2007; Vedel‐Sørensen et al., 2013).

*Butia yatay* (Mart.) Becc. is endemic to subtropical South America in areas with a warm–rainy climate, where it thrives mostly on sandy and acidic soils (Carnevali, 1994; Martinez Crovetto & Piccinini, 1950). It grows in palm groves and displays complex spatial patterns (Batista et al., 2014; Marcato, 2004). Fossil records, based on phytoliths, link these palm communities to Upper Pleistocene formations of the Uruguay and Paraná Rivers (Patterer & Zucol, 2014; Patterer et al., 2017, 2019). The phytoliths of these formations are derived from a humid savanna ecosystem with meso‐megathermic grasslands and open forests (Contreras et al., 2019; Erra et al., 2013; Patterer et al., 2019).

The taxonomic circumscription and distribution of *B. yatay* have been the subject of some controversy. We consider the palm groves in Argentina and Uruguay as natural populations of *B. yatay* and align with Deble et al. (2011, 2012a) and Noblick (2014), who differentiate the palm trees observed in Brazil (Soares, 2015) as distinct from *B. yatay*. It is worth mentioning that *Butia noblickii* Deble, Marchiori, F.S. Alves & A.S. Oliveira (Deble et al., 2012b), *Butia paraguayensis* (Barb. Rodr.) L.H. Bailey (Noblick, 2014), and *Butia poni* (Haumann) Burret. (Deble et al., 2017) all occur in Argentina and are distinct species from *B. yatay*.

Here, we assessed whether the geographical distribution of *B. yatay* changed over time and whether its past distribution is consistent with the Pleistocene refugia proposed for the South America flora (Whitmore & Prance, 1987) or fossil records of this species dated to the Upper Pleistocene (Patterer & Zucol, 2014; Patterer et al., 2017, 2019). Additionally, we explored which variables determine the present distribution of *B. yatay*, using two approaches: bioclimatic‐only and bioclimatic with nonclimatic variables. For this work, we performed species distribution modeling (SDM), which employs machine learning and statistical methods to link species presence data with bioclimatic and nonclimatic variables. Moreover, this modeling enabled us to predict suitable habitats for species occurrence over time (Phillips et al., 2006) and to unravel their evolutionary history (Henrot et al., 2017). Incorporating nonclimatic variables, such as soil and topographic factors, into SDMs supports more accurate determination of plant species distribution (Chozas et al., 2017; Velazco et al., 2017). Therefore, we performed SDM using bioclimatic and nonclimatic variables to (1) estimate the habitat suitability of *B. yatay* in the present using two different approaches, (2) evaluate the importance of soil variables for the present distribution, and (3) predict historical ranges by determining correspondences with proposed Pleistocene refugia and fossil records. Based on previous findings of northward shifts in subtropical vegetation during the glacial period in the Chaco, we hypothesized that *B. yatay* also underwent a range shift toward the north. In addition, we hypothesized that despite climate being a primary factor influencing plant distribution in the Chaco, edaphic variables also play a significant role at the local scale and could enhance the accuracy of the modeled distribution of *B. yatay*. For this reason, when this information is available, these variables should be included in models.

## 2 MATERIALS AND METHODS

### 2.1 Study area

**FIGURE 1:**
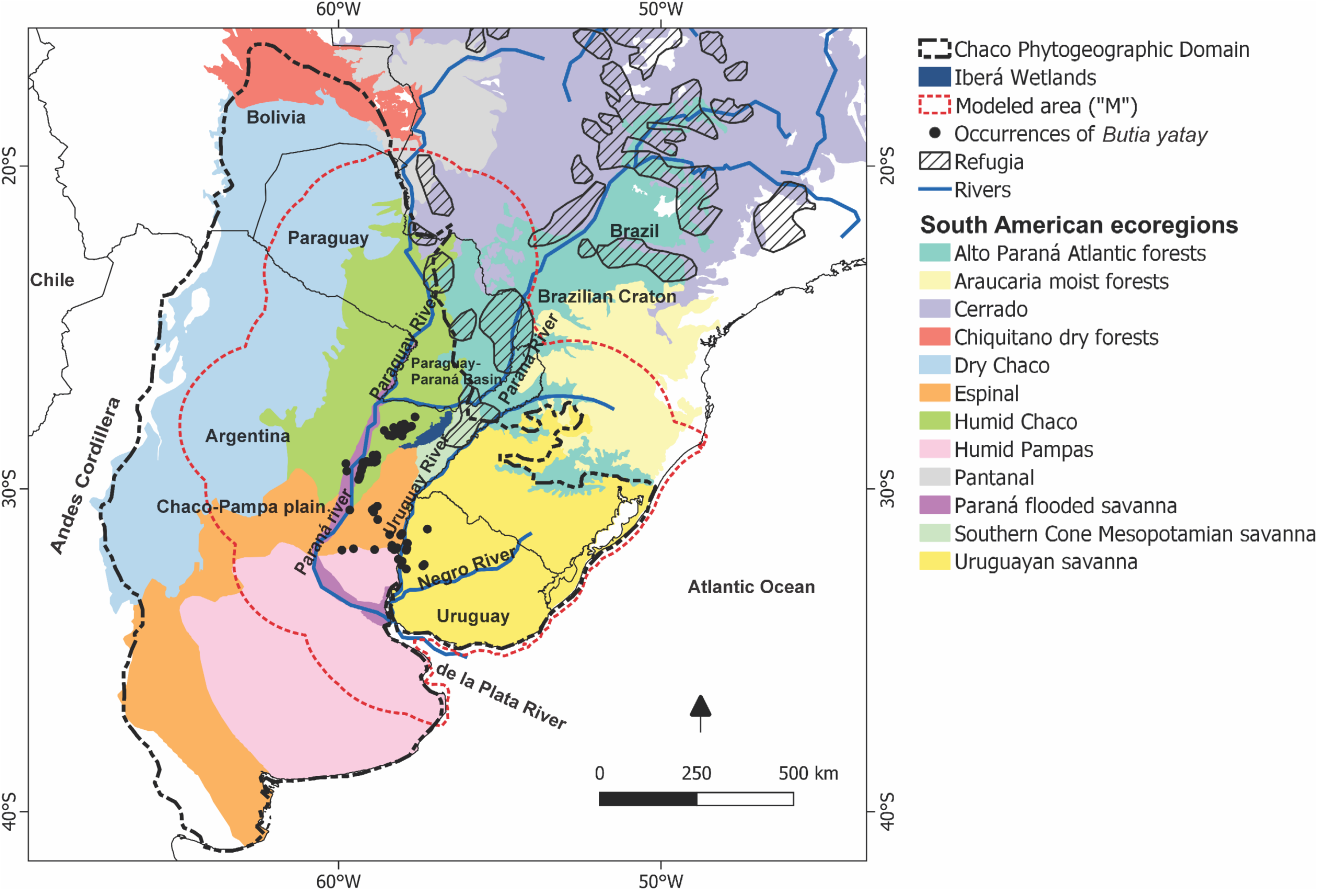
Present distribution of *Butia yatay* in the Humid Chaco, Espinal, and Uruguayan savanna ecoregions. The red stippled line surrounds the area used for environmental niche modeling (modeled area “M”). The black stippled line shows the boundary of the Chaco Phytogeographic Domain (sensu Cabrera & Willink, 1973). The distribution of ecoregions is based on Olson et al. (2001), and the occurrences of *B. yatay* are based on our own research. Striped polygons showing the occurrence of Pleistocene refugia were redrawn from Whitmore and Prance (1987).

Our research is focused on the Chaco Phytogeographic Domain (Chaco; Cabrera & Willink, 1973). The study area mainly covers the Humid Chaco, Espinal, and Uruguayan savanna ecoregions (Olson et al., 2001; Figure 1). These ecoregions exhibit a subtropical climate with precipitation and temperature gradients spanning west to east and north to south (Morello et al., 2012). The mean annual temperature hovers around 23–24°C, and the annual precipitation range is 750–1300 mm (Morello et al., 2012). The region's soils display notable diversity and complexity, characterized by deep horizons rich in organic matter and slight acidity. Moreover, the soils have low cation exchange capacity (CEC) because of the very low‐activity clays. The topography is flat, ranging in elevation from sea level up to about 500 m. Notably, lowlands frequently experience flooding, and permanent lakes and ponds dot the landscape, typified by the Iberá wetlands and the areas adjacent to the Paraná River (Navarro de Rau, 2019). The vegetation is a mosaic of forest, grasslands, and savannas or exclusively savannas in the southeast (Oyarzabal et al., 2018). Morphogenetic and climatic changes have influenced the development of the Gran Chaco landscape. Tectonic movements and active faulting have played a pivotal role in shaping the courses of rivers, particularly the Paraná and Uruguay Rivers. Over time, these rivers have undergone complex ontogeny, altering their paths from the Tertiary to their present configurations (Popolizio, 2006). Additionally, during drier Pleistocene periods, significant eolian activity has contributed to the erosion and deposition of substantial amounts of silt and sand across the region (Iriondo & Garcia, 1993).

### 2.2 Data

#### 2.2.1 Palm records

We compiled 110 records for *B. yatay* from our field observations during October 2019 and georeferenced data from the GBIF database (http://www.gbif.org, GBIF, 2022), SpeciesLink (http://splink.cria.org.br, SpeciesLink, 2022), and the herbarium of the Instituto de Botánica del Nordeste (CTES). After conducting a rigorous check of all occurrences and removing geographic outliers (i.e., urban locations assumed to involve cultivated individuals and duplicates), we retained sites over 5 km apart to reduce spatial autocorrelation bias, averting overrepresentation that influences habitat suitability (Hijmans et al., 2017; Phillips et al., 2009). The final dataset included 69 occurrences for *B. yatay* (Figure 1; Appendix S1).

#### 2.2.2 Environmental data

We used 19 bioclimatic variables from the CHELSA 2.1 database with a spatial resolution of 30 arcsec (Karger et al., 2021). Additionally, global slope data from the EarthEnv project, with a resolution of 30 arcsec, were incorporated (https://www.earthenv.org, Amatulli et al., 2018). We also included five soil variables at 250 m resolution (CEC, clay content, pH water, sand content, and silt content) obtained from the ISRIC‐World Soil Information Database 2.0 (http://soilgrids.org, Poggio et al., 2021; Appendix S2). To manage soil data, we calculated weighted averages across six depths (0–5, 5–15, 15–30, 30–60, 60–100, and 100–200 cm) to generate a unified value. Subsequently, these data were resampled to 30 arcsec resolution using the R “Raster” package (Hijmans, 2016). The selection of these specific soil variables was grounded in their significance in determining palm species distributions (Eiserhardt et al., 2011), as well as their local influence on *B. yatay* distribution (Martinez Crovetto & Piccinini, 1950; Parques Nacionales, 2023). All 25 variables were extracted for the modeled area (“M” area in Figure 1) around the Humid Chaco, Espinal, and Uruguayan savanna ecoregions with a buffer zone of 250 km, as suggested by Barve et al. (2011) and Soberon and Peterson (2005).

### 2.3 Analysis

#### 2.3.1 Species distribution modeling

To reduce collinearity and potential overfitting of the model, we assessed collinearity among all predictors using Pearson correlations (Dormann et al., 2013) and calculated among all variables in the original set of 25 environmental predictors. We excluded one of the variables for every pair of variables in which the correlation coefficients (*r*) were > .7.

The final models included five bioclimatic variables: mean annual air temperature (Bio 1), mean diurnal air temperature range (Bio 2), mean daily mean air temperatures of the wettest quarter (Bio 8), mean daily mean air temperatures of the driest quarter (Bio 9), and precipitation amount in the driest month (Bio 14). Additionally, global slope data and three soil variables were included: CEC, clay content, and pH water (pH; Appendix S3). For each of these variables, information was extracted from the points of occurrence of *B. yatay* to determine the current ranges of environmental preference.

Based on the selected variables, we used two groups of layers to determine the geographic distribution of suitable habitat for *B. yatay* in the present (1989–2010). One group exclusively consisted of bioclimatic variables, and the other incorporated soil and globe slope variables in addition to the bioclimatic variables. We employed two machine learning models, MaxEnt 3.4.4. and Random Forest 4.7.1.1. The aim of this approach was to minimize uncertainties stemming from varying climate models. These algorithms are among the top performers for presence–background modeling across geographical contexts and scales of analysis and with different species (Phillips et al., 2006; Valavi et al., 2021, 2022).

For the MaxEnt model, we adjusted specific parameters to improve model performance and robustness to sampling bias (Warren et al., 2014) using the R “ENMeval 2.0” package (Kass et al., 2021). Specifically, we developed feature class and regularization parameters that regulate model complexity using five random k‐folds. We created 24 models by combining feature classes (linear, product, quadratic, and hinge) with a range of regularization multipliers (i.e., the penalty for including additional constraints in the model) from 0.5 to 2.0 in increments of 0.5. The combination of settings with the highest value of the area under the curve (AUC) was selected as the optimal model (Kass et al., 2021; Velasco & González‐Salazar, 2019; Appendix S4).

In the random forest (RF) model, we used the RF downsample setting because it outperforms the default RF approach (Valavi et al., 2021, 2022). The RF models were fitted with 1000 trees, each constructed from a bootstrap sample containing both presence and an equivalent number of background points. To build the model, we used the R “randomForest” package (Breiman et al., 2018).

To analyze the importance of each environmental variable, we used the percent contribution for MaxEnt models (Phillips et al., 2006) and mean decrease Gini (MDG) in RF models (Breiman, 2018; Nicodemus, 2011). Both metrics indicate the extent to which each environmental variable influences the performance of the model.

To evaluate the predictive performance of the models, we used the area under the receiver operating characteristic curve (i.e., AUC) and the true skill statistic (TSS; Allouche et al., 2006; Phillips et al., 2006). We computed these metrics using the R “Biomod2” (Thuiller et al., 2016) and R “PRoc” packages (Robin et al., 2011). AUC values >0.7 indicate that the model has high accuracy in the prediction (Peterson et al., 2011). TSS values range from ‐1 to 1, and TSS values >0.6 are considered good models (Allouche et al., 2006; Thuiller et al., 2016).

To calculate the predicted suitable area for distribution models, we generated binary models applying the threshold maximum training sensitivity plus specificity recommended for models with presence‐only data (Liu et al., 2016). We performed all analyses using the R environment version 4.1 (R Development Core Team, 2019).

#### 2.3.2 Historical distribution

To predict the past distribution for *B. yatay*, we used historical bioclimatic variables from the PaleoClim database at 2.5 arcmin of resolution (Brown et al., 2018). Models have been built on present climate conditions and then projected to the Marine Isotope Stage 19 (MIS 19; ca. 787 ka), Last Interglacial (LIG; ca. 130 ka), LGM (ca. 21 ka), Heinrich Stadial 1 (HS; ca. 17.0–14.7 ka), Bølling‐Allerød (BA; 14.7–12.9 ka), Younger Dryas Stadial (YDS; 12.9–11.7 ka), Early Holocene (EH; ca. 11.7–8.326 ka), Mid‐Holocene (MH; 8.326–4.2 ka), and Late Holocene (LH; ca. 4.2–0.3 ka). Furthermore, to identify climate extrapolation across different past periods, we employed multivariate environmental similarity surfaces (MESS) analysis (Elith et al., 2010) through the R “Dismo” package (Hijmans et al., 2017). MESS analyses assess the environmental similarity of variables and identify regions where one or more environmental variables fall outside of the training range. Negative values indicate areas where at least one variable has a value outside the range of values, suggesting a novel predicted environment. Conversely, positive values reflect similarities between variables from different time periods and the present variables, and higher positive values correspond to a closer resemblance to the present conditions (Elith et al., 2010). To assess whether the areas where *B. yatay* potentially existed in the past align with suggested Pleistocene refugia for South America flora (Whitmore & Prance, 1987), we overlapped the Pleistocene refugia layer with the LGM distribution maps. In addition, we compared predicted past distribution to known occurrences in the Upper Pleistocene fossil record by testing the overlap of past modeled distributions and the phytolith‐based fossil record (Patterer & Zucol, 2014; Patterer et al., 2017, 2019).

## 3 RESULTS

### 3.1 Species distribution models

**FIGURE 2:**
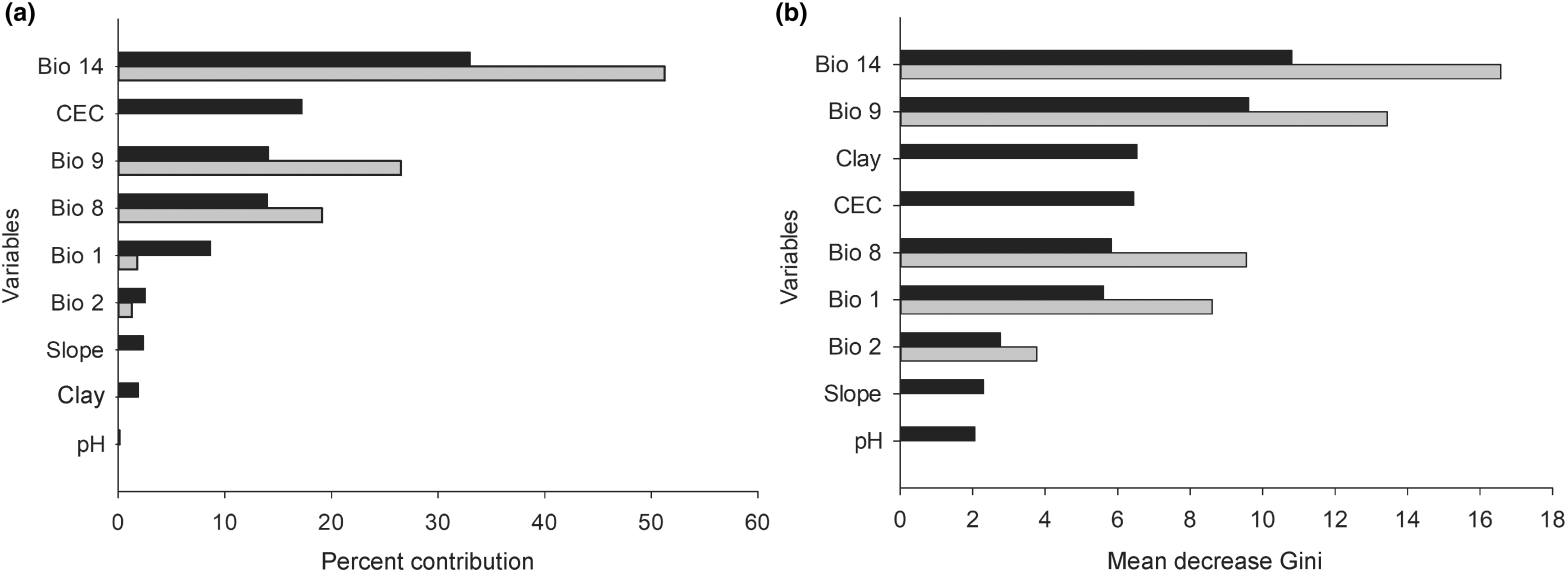
Relative contribution and MDG of each variable used for species distribution models of *Butia yatay* in the present using (a) MaxEnt and (b) RF. Gray bars represent variables in the model of present climate‐only, and black bars represent variables in the model of present climate, topographic, and soil.

The relative contribution for the MaxEnt model and MDG of each variable for RF in the present models is shown in Figure 2. Mean values and range of variation for each variable used in the *B. yatay* present models are displayed in Table 1.

**TABLE 1:** Variation range and mean values extracted from bioclimatic, soil, and topographic variables, providing insights into the environmental preferences of *B. yatay.*

Variables	Abbreviation	Unit	Mean	Minimum–maximum
Mean annual air temperature	Bio 1	°C*10	19.70	17.85–21.35
Mean diurnal air temperature range	Bio 2	°C*10	9.17	8.70–9.70
Mean daily mean air temperatures of the wettest quarter	Bio 8	°C*10	22.73	21.20–23.95
Mean daily mean air temperatures of the driest quarter	Bio 9	°C*10	14.02	11.85–16.04
Precipitation amount of the driest month	Bio 14	mm/month	44.00	29.60–69.40
Cation exchange capacity (at pH 7)	CEC	mmol(c)/kg	231.45	195.00–391.00
Clay content	Clay	g/kg	241.02	73.40–440.00
pH water	pH	pH*10	4.30	3.70–5.20
Slope	Slope	Degree	0.80	0.22–27.49

**FIGURE 3:**
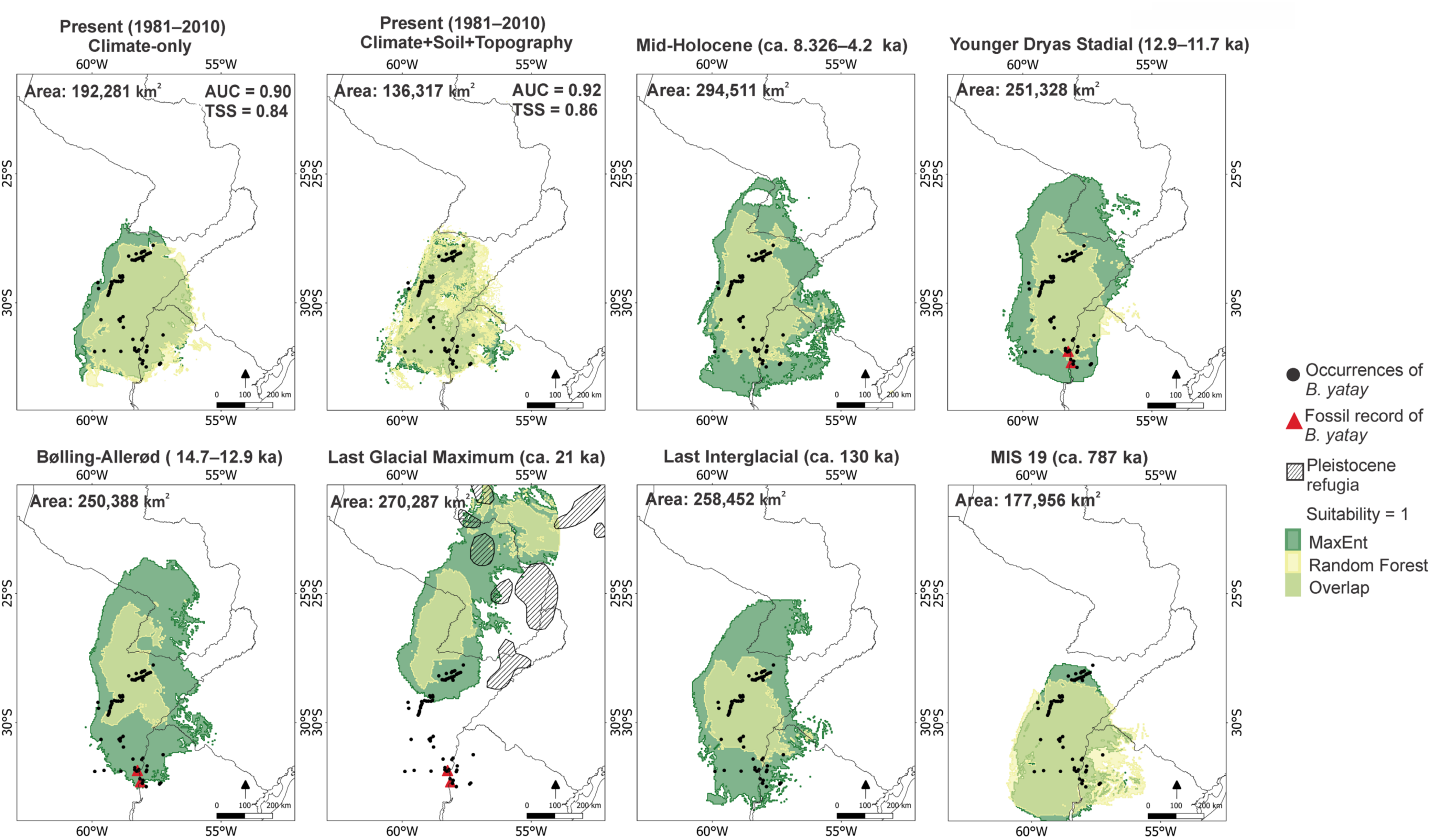
Predicted distribution of suitable habitats for *Butia yatay* under present and past climatic conditions using the MaxEnt and RF algorithms and their overlap. Striped polygons in the LGM map show the occurrence of Pleistocene refugia, redrawn from Whitmore and Prance (1987). The red triangles show fossil records found in sediments from the Upper Pleistocene (ca. 11–120 ka; Patterer & Zucol, 2014; Patterer et al., 2017, 2019).

Evaluation using AUC (0.9) and TSS (0.8) showed that both models performed well in predicting species distribution (Figure 3). Under present conditions, the range of suitable areas was similar between MaxEnt and RF, covering the known and recorded locations of the palm tree. The overall prediction area was continuous across the southeastern Humid Chaco, northern Humid Pampa, Espinal, Southern Cone Mesopotamian savanna, and northeastern Uruguayan savanna (Figure 3). Incorporating soil variables into both models resulted in a ~30% reduction in the suitability area, and accuracy increased by 0.02 (Figure 3). The climate‐only model indicated suitable conditions in the northwest, covering the Southern Cone Mesopotamian savanna and Espinal ecoregions. However, when climate and soil were considered together, the analysis suggested that edaphic variables might limit habitat suitability in those particular areas. Therefore, the model aligned more closely with the actual distribution of the species.

### 3.2 Historical distribution

From the paleoclimate projections (Figure 3), we show results from scenarios with the greatest changes in the distribution range and from the Upper Pleistocene, where the fossil data were discovered (in strata from the Upper Pleistocene Formation; for other projections, see Appendix S5). The suitable areas in the MIS 19 (ca. 787 ka), LIG (ca. 130 ka), and MH (8.326–4.2 ka) are congruent with ecoregions where *B. yatay* is currently distributed. Additionally, the models suggested that during LIG and HS (ca. 17.0–14.7 ka), *B. yatay* experienced a significant contraction in distribution range before and after the LGM (ca. 21 ka), respectively. However, in the LGM and Holocene, the suitable area for *B. yatay* may have expanded. The LGM model showed significant distribution shifts, indicating that the suitability area moved northward. The palm could have inhabited areas that are now the southeastern Cerrado, the eastern Alto Paraná Atlantic Forest, the northern Humid Chaco and Espinal, and the southern part of the Southern Cone Mesopotamian savanna. The geographic distribution of suitable habitat during the LGM overlapped with a few Pleistocene refugia located further south but not with the fossil occurrence. The location of phytolith‐based fossil records from the Upper Pleistocene (126–11.7 ka) overlapped with the geographic distribution of suitable habitat during the YDS (12.9–11.7 ka) in the RF model, whereas in the MaxEnt model, the overlap was with the BA (14.7–12.9 ka) and YDS, that is, 14.7–11.7 ka.

The MESS analysis for *B. yatay* (Appendix S6) identified multiple areas where no analogue or novel climates were present, predominantly in the eastern zone of the designated “M” area. However, our models found these areas outside their geographic suitability area.

## 4 DISCUSSION

The biogeographical changes in the subtropical flora of South America during the Quaternary constitute an important knowledge gap. This research shows the influence of precipitation and temperature in the driest month on the climate preferences of the subtropical endemic palm *B. yatay*. Moreover, we highlight the importance of soil variables for plant distribution and ecology. During the Quaternary, *B. yatay* distribution could have shifted because of climatic fluctuations. These changes may have forced the colonization of new areas and persisted in specific northern regions during unfavorable periods. Our findings contribute to knowledge about changes in the flora of the Chaco, providing valuable insights for future research on the Arecaceae (palms) and other taxa within this region.

### 4.1 Species distribution models

Explaining the observed distribution and ecological preferences of species is one of the central goals in ecology (Krebs, 2009). *Butia yatay* distribution patterns reflect interactions among environmental variables, including bioclimatic features, soils, and topographic conditions. Using MaxEnt and RF algorithms for SDM, we generated robust predictions supported by high AUC and TSS values. The present suitable habitat for *B. yatay* would cover ~160,000 km^2^, primarily within Chaco's central ecoregions, bordered by the Paraná, Uruguay, and Negro Rivers. The niche preferences of *B. yatay* include sandy hills with slopes ranging from 0.22 to 27 degrees, acidic soils with low CEC because of very low‐activity clays, and climate conditions (temperature and precipitation) resembling those reported by Martinez Crovetto and Piccinini in 1950. These results suggest that species have maintained these preferences over the past 70 years. Our research also provides novel insights into additional environmental preferences, such as slope, CEC, and clay content for *B. yatay*.

Our findings align with previous continental‐scale palm studies and highlight the significance of precipitation in the driest month (Bio 14) and mean daily air temperatures in the driest quarter (Bio 9) as primary influencing variables. These factors consistently emerge as pivotal in shaping palm distribution (Balslev et al., 2011; Eiserhardt et al., 2011; Vedel‐Sørensen et al., 2013), with precipitation recognized as a key ecological factor for Chaco taxa distribution (Rezende et al., 2020). Additionally, our research underscores the importance of temperature variability, particularly seasonally, over other temperature‐related metrics such as mean annual temperature (Bio 2). This emphasis arises from the common constraint on plant species distributions by temperature fluctuations and extremes (Silva de Miranda et al., 2018), a pattern observed globally, including for South American palms (Vedel‐Sørensen et al., 2013; Zuquim et al., 2020).

Although the climate is usually considered the main driver of plant distributions (Silva de Miranda et al., 2018), our study indicates that the distribution of *B. yatay* also could be strongly influenced by CEC and clay content. These findings are in agreement with other studies on palms in the Americas, which also have identified soil characteristics as limiting factors for palm distribution (Eiserhardt et al., 2011; Vedel‐Sørensen et al., 2013). The topographic variable slope shows a low influence on the models, however, and the degree of topographic influence on palm distributions can vary among species. In some cases, it has a strong, slight, or zero influence on distribution (Balslev et al., 2011; Vormisto et al., 2004). This variability may be attributed to the indirect nature of topography's influence, which often operates through its correlation with other environmental factors, such as soil drainage patterns (Kahn, 1987).

Climate significantly influences soil formation and various edaphic processes and properties (Walthert & Meier, 2017), but the correlation of these factors is not strong enough to exclude them from SDMs. Moreover, climate‐only models are conceptually weak, and the inclusion of soil and topographic variables improved the performance of SDMs in the current work. Incorporating this set of variables led to a projection of the present habitat that more closely approximated the actual distribution compared with using the model incorporating climate variables only. These results reinforce the widely accepted idea that soil variables are among the most important factors affecting plant distribution (Solís Neffa et al., 2022; Velazco et al., 2017; Zuquim et al., 2020). Indeed, soil properties control the distribution of plant species where climatic conditions can be very homogenous (Diekmann et al., 2015; Velazco et al., 2017) and represent a key factor for diversification and spatial segregation of parapatric species (Chozas et al., 2017). These findings highlight the importance of soil in the SDMs and of incorporating soil features into these models. For future work, soil data at a finer scale could provide more refined results because of the heterogeneity of soils in the region, which would further enhance the precision of environmental preferences. The availability of data, such as soil aluminum and phosphorus content, that are relevant for palm trees will improve the prediction capability of the models.

### 4.2 Historical distribution modeling

The evolutionary processes that have shaped the unique biodiversity of the Chaco remain unexplored (Rull & Carnaval, 2020). We must exercise caution in interpreting data outside the present climate range (Carneiro et al., 2016), but paleomodeling has demonstrated its significance as a predictor of regional, continental, and global palm distribution patterns (Bueno et al., 2017; da Silva Carvalho et al., 2017; de Lima et al., 2018; Kissling et al., 2012; Tovaranonte et al., 2015). In this study, we present the first examination of the potential effects of Quaternary climatic fluctuations on the biogeographical history of the subtropical palm tree *B. yatay*.

Quaternary landscapes in this part of Chaco were mainly open biomes of grassland with palms growing along river margins (Contreras et al., 2019; Erra et al., 2013). The paleodistribution analysis suggested that the glacial/interglacial cycles could have affected the distribution of *B. yatay*. The predicted suitable range during MIS 19 could have covered continuous areas within the same ecoregions as the present (~187,113 km^2^). A recent study focusing on the herb *Turnera sidoides* L. in the Chaco, similarly identified suitable areas during MIS 19 and the present within the same ecoregions (Solís Neffa et al., 2022). These findings provide support for the concept that among the older interglacial periods, the MIS 19 closely resembled the present (Regattieri et al., 2019).

In the subsequent evolutionary history of *B. yatay* during the LIG, suitable areas (~176,900 km^2^) may have decreased compared with the MIS 19 and LGM periods. The LIG has been considered warmer and wetter than the present (Iriondo, 2010). Conversely, the LGM, characterized by dry and cold conditions, would have resulted in a northward shift of the subtropical climate (Ab'Sáber, 1977; Iriondo, 2010). Our model suggested the first expansion of the suitable area (~191,900 km^2^) during this period. The only other study that has examined a subtropical palm (*Euterpe edulis* Mart.) supports that there was a palm expansion during the LGM, reinforcing our findings (da Silva Carvalho et al., 2017).

*Butia yatay* could have shifted northward, occupying new areas along the southeastern Cerrado, the northern parts of the Humid Chaco, the eastern Alto Paraná Atlantic Forest, the north of the Espinal, and the southern parts of the Southern Cone Mesopotamian savanna. This distribution might have coincided with some of the Pleistocene refugia located in the south (Whitmore & Prance, 1987), between the Paraná and Paraguay Rivers. During this period, the River channels were wider and seasonally dry during glacial intervals (Clapperton, 1993), potentially serving as refugia for flora, including *B. yatay*, that was adapted to humid climates (Ab'Sáber, 1977). The LGM paleodistribution of suitable habitat of Chaco tree species, which are currently distributed along the Paraná River, also has indicated a slight northward expansion (Giudicelli et al., 2019; Sinani et al., 2022; Spichiger et al., 2004). Therefore, we can infer that this area would have provided favorable conditions for the Chaco flora during the climatic fluctuations of the LGM. Moreover, this finding implies the great influence of the Paraná River on the present and past distributions of both *B. yatay* and other Chaco species.

An alternative perspective on the LGM suggests that plant communities within the Chaco may have persisted. The relative abundance of these species could have varied in response to different fluvial facies of the Paraná and Uruguay Rivers (Erra et al., 2013). However, in relation to both hypotheses, our results align with the notion that both the LIG and LGM periods are consistent with the predicted expansion of the South American savanna, followed by its contraction (Ab'Sáber, 1977; Haffer, 1969; Hewitt, 2000; Whitmore & Prance, 1987). In this context, analyses using a larger sample of Chaco species may reveal a more consistent trend in support of one of the hypotheses because overall patterns could be less affected by the particular ecological and life‐history traits of a species. Even so, the distinct responses of individual species that offer support for a hypothesis are important (Collevatti et al., 2013).

According to fossil‐phytolith evidence, *B. yatay* was present in the Palmar Formation during the Upper Pleistocene (126–11.7 ka). These sediments represent braided river deposits formed in two cycles, indicating changes in the vegetation composition of the paleocommunity (Patterer et al., 2017). The first cycle may indicate a change in the physiognomy of the landscape and the retraction of *B. yatay* (Patterer et al., 2017). The second cycle suggests the presence of palms with meso‐megathermic grassland in a humid subtropical‐tropical climate (Patterer & Zucol, 2014; Patterer et al., 2020). The overlap of fossil‐phytolith data and paleoclimate models from the Upper Pleistocene suggests a potential alignment with *B. yatay'*s suitable habitat during the BA and YDS periods but not during the HS and LGM. Both results may indicate a change in vegetation in the Upper Pleistocene, possibly coinciding with *B. yatay* recolonization of the area following the BA. Pollen accumulation rates in the BA and YDS periods suggest that the YDS did not exist as such in the northern hemisphere for the mid‐latitudes in South America (Markgraf, 1991). The distribution of the species does not appear to have shifted, but population densities may have been lower. This finding aligns with the observation that *B. yatay* distribution may have remained almost unchanged during the same periods. Overall, these results make a significant contribution by highlighting BA as the probable period in the Upper Pleistocene during which the phytoliths of *B. yatay* were deposited, providing valuable insights into its historical distribution.

The models suggest that in the transition to the Holocene, *B. yatay* may have experienced a second expansion (~215,954 km^2^). Several studies of tropical palms indicate that savannas expanded in the transition to the Holocene and not in the LGM (Bueno et al., 2017; de Lima et al., 2018). However, an alternative view proposed by Mayle et al. (2004) is that the expansion occurred in both periods, which aligns with our findings.

Even though SDMs and the fossil record are highly valued, they have limitations. Our paleoclimate projections assumed unrestricted dispersal models and did not consider potential biogeographical barriers. Incorporating dispersal processes and biotic interactions into SDMs could better distinguish accessible and inaccessible suitable areas. This differentiation is crucial for achieving more precise projections of range shifts (Uribe‐Rivera et al., 2017). Another limitation of SDMs is the absence of edaphic and topographic grids for past climates. Despite their availability for extensive and varied regions in contemporary times (Amatulli et al., 2018; Poggio et al., 2021), their extension to past climatic conditions remain a pending endeavor. Furthermore, the fossil record for *B. yatay* is sparse, and supplementary fossil data might have been derived from the presence of sister taxa, although fossil records of the genus *Butia* are notably scant. In this sense, despite our LGM model being unable to identify “true refugia”—that is, areas where a species persisted in unfavorable climatic conditions—it suggested likely past areas of distribution. In turn, the results have raised a fresh set of challenges and inquiries that require further exploration. Future paleontological and molecular biogeographic approaches will be necessary to rigorously test these models in the future.

## 5 CONCLUSIONS

The current distribution of *B. yatay* is likely driven by precipitation and temperature during the driest month, alongside soil variables such as CEC and clay content. The model incorporating both bioclimatic and nonclimatic variables highlights the importance of soil in shaping the distribution and ecological dynamics of *B. yatay* in the present. Nevertheless, a variety of relevant environmental factors must be considered, both in the current context and across broader temporal scales. Dispersion patterns and availability of nitrogen and aluminum, among other factors, are crucial to consider and must be integrated into future research models.

During the Quaternary, *B. yatay* may have undergone dynamic fluctuations in its range, involving localized extinctions followed by partial recolonization in northern regions during the last glacial period. Within this timeframe, it is plausible that the species inhabited specific Pleistocene refugia along the Paraguay and Paraná Rivers. The relationship between *B. yatay* and the Paraguay‐Paraná basin during the LGM suggests that river geomorphology significantly influenced its distribution. Furthermore, the presence of phytoliths in sediment samples closely correlates with the dynamics of adjacent rivers. The combination of paleomodeling and phytolith records from Pleistocene sediments may indicate that these phytoliths were deposited approximately 12–14 ka.

These findings highlight the myriad factors shaping the distribution and ecology of *B. yatay*. Moreover, they contribute to an understanding of shifts in subtropical flora during the Quaternary and provide an approach that may be useful for the study of other taxa in the Chaco Phytogeographic Domain.

## AUTHOR CONTRIBUTIONS

**G. Carolina Silva:** Conceptualization (equal); formal analysis (lead); investigation (equal); methodology (lead); validation (equal); visualization (lead); writing – original draft (lead); writing – review and editing (equal). **Viviana Griselda Solís Neffa:** Conceptualization (lead); investigation (lead); supervision (lead); writing – review and editing (lead). **Gabriela Zuquim:** Formal analysis (equal); investigation (equal); methodology (equal); validation (equal); writing – review and editing (equal). **Henrik Balslev:** Conceptualization (lead); funding acquisition (lead); investigation (equal); project administration (lead); resources (lead); supervision (lead); writing – review and editing (lead).

## CONFLICT OF INTEREST STATEMENT

The authors declare that they have no conflicts of interest to report.

## Supporting information


Appendix S1

## Data Availability

Data Availability Statement is included in supplementary material provided in the supporting information file.
