# Supplementary material for: Biogeography and environmental preferences of Butia yatay (Mart.) Becc
Source: Ecol Evol. 2023 Nov 27;13(11):e10749. doi: 10.1002/ece3.10749 (PMC10682568; doi:10.1002/ece3.10749)

**SUPPORTING INFORMATION**

**Appendix S1:** Occurrence data for *Butia yatay* used in developing the final distribution models.

| ID | Longitude | Latitude |  | ID | Longitude | Latitude |
| --- | --- | --- | --- | --- | --- | --- |
| 1 | -57.63 | -27.77 |  | 37 | -59.29 | -29.43 |
| 2 | -57.96 | -27.99 |  | 38 | -59.74 | -29.44 |
| 3 | -58.00 | -28.01 |  | 39 | -59.33 | -29.55 |
| 4 | -58.06 | -28.02 |  | 40 | -59.33 | -29.60 |
| 5 | -57.73 | -28.05 |  | 41 | -59.35 | -29.66 |
| 6 | -58.09 | -28.06 |  | 42 | -59.38 | -29.71 |
| 7 | -57.79 | -28.08 |  | 43 | -58.79 | -30.53 |
| 8 | -58.29 | -28.10 |  | 44 | -58.89 | -30.61 |
| 9 | -57.85 | -28.10 |  | 45 | -58.95 | -30.65 |
| 10 | -57.89 | -28.12 |  | 46 | -59.65 | -30.65 |
| 11 | -58.58 | -28.19 |  | 47 | -58.90 | -30.71 |
| 12 | -57.99 | -28.20 |  | 48 | -58.78 | -30.95 |
| 13 | -58.04 | -28.22 |  | 49 | -57.24 | -31.24 |
| 14 | -58.09 | -28.24 |  | 50 | -58.03 | -31.38 |
| 15 | -58.18 | -28.27 |  | 51 | -58.41 | -31.41 |
| 16 | -58.05 | -28.28 |  | 52 | -58.07 | -31.43 |
| 17 | -58.23 | -28.30 |  | 53 | -57.86 | -31.68 |
| 18 | -58.28 | -28.31 |  | 54 | -58.34 | -31.74 |
| 19 | -58.45 | -28.33 |  | 55 | -58.18 | -31.82 |
| 20 | -58.38 | -28.34 |  | 56 | -58.34 | -31.82 |
| 21 | -58.32 | -28.35 |  | 57 | -57.88 | -31.84 |
| 22 | -58.03 | -28.36 |  | 58 | -59.53 | -31.85 |
| 23 | -58.82 | -28.95 |  | 59 | -58.21 | -31.86 |
| 24 | -58.93 | -28.96 |  | 60 | -58.88 | -31.88 |
| 25 | -58.81 | -29.00 |  | 61 | -58.27 | -31.88 |
| 26 | -58.90 | -29.01 |  | 62 | -59.89 | -31.89 |
| 27 | -58.82 | -29.14 |  | 63 | -57.90 | -31.90 |
| 28 | -59.13 | -29.14 |  | 64 | -58.14 | -32.17 |
| 29 | -58.98 | -29.17 |  | 65 | -58.02 | -32.24 |
| 30 | -59.05 | -29.17 |  | 66 | -57.34 | -32.34 |
| 31 | -58.88 | -29.17 |  | 67 | -58.04 | -32.35 |
| 32 | -59.24 | -29.18 |  | 68 | -57.38 | -32.38 |
| 33 | -59.77 | -29.21 |  | 69 | -57.90 | -32.48 |
| 34 | -59.22 | -29.25 |  |  |  |  |
| 35 | -59.25 | -29.31 |  |  |  |  |
| 36 | -59.28 | -29.37 |  |  |  |  |

**Appendix S2:** Environmental variables used in modeling the spatial distribution of *B. yatay*.

| Abbreviation | Long Name | Unit | Data Base |
| --- | --- | --- | --- |
| Bio 1 | Mean annual air temperature | °C*10 | CHELSA |
| Bio 2 | Mean diurnal air temperature range | °C*10 | CHELSA |
| Bio 3 | Isothermality | °C | CHELSA |
| Bio 4 | Temperature seasonality | °C*10 | CHELSA |
| Bio 5 | Mean daily maximum air temperature of the warmest month | °C*10 | CHELSA |
| Bio 6 | Mean daily minimum air temperature of the coldest month | °C*10 | CHELSA |
| Bio 7 | Annual range of air temperature | °C*10 | CHELSA |
| Bio 8 | Mean daily mean air temperatures of the wettest quarter | °C*10 | CHELSA |
| Bio 9 | Mean daily mean air temperatures of the driest quarter | °C*10 | CHELSA |
| Bio 10 | Mean daily mean air temperatures of the warmest quarter | °C*10 | CHELSA |
| Bio 11 | Mean daily mean air temperatures of the coldest quarter | °C*10 | CHELSA |
| Bio 12 | Annual precipitation amount | mm/year | CHELSA |
| Bio 13 | Precipitation amount of the wettest month | mm/month | CHELSA |
| Bio 14 | Precipitation amount of the driest month | mm/month | CHELSA |
| Bio 15 | Precipitation seasonality | mm | CHELSA |
| Bio 16 | Mean monthly precipitation amount of the wettest quarter | mm/quarter | CHELSA |
| Bio 17 | Mean monthly precipitation amount of the driest quarter | mm/quarter | CHELSA |
| Bio 18 | Mean monthly precipitation amount of the warmest quarter | mm/quarter | CHELSA |
| Bio 19 | Mean monthly precipitation amount of the coldest quarter | mm/quarter | CHELSA |
| CEC | Cation exchange capacity (at pH 7) | mmol(c)/kg | Soil Grid |
| Clay | Clay content | g/kg | Soil Grid |
| Slope | Slope | m | Earth Env |
| pH | pH water | pH*10 | Soil Grid |
| SAND | Sand content | g/kg | Soil Grid |
| SILT | Silt content | g/kg | Soil Grid |

**Appendix S3:** Pearson correlation among bioclimatic, soil, and topographic variables used in the SDM. All variables for which correlation coefficients were >0.70 were excluded.


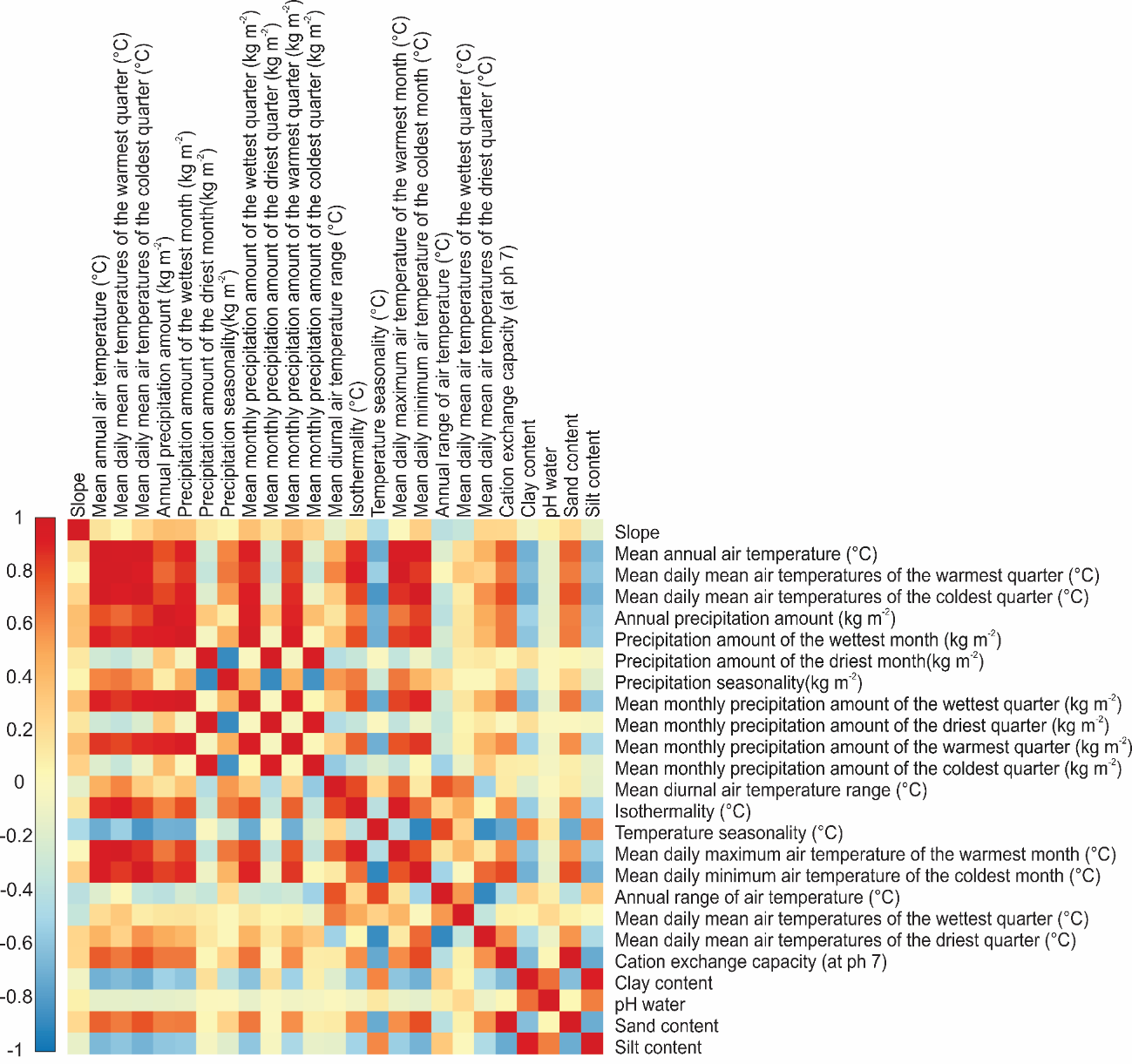


**Appendix** **S4**: Details of the models based on present conditions for *Butia yatay* performed with the maximum entropy algorithm (MaxEnt). Feature **(FC)** refers to the mathematical transformations of covariates that the model applies to allow complex relationships: linear **(L)**, linear quadratic **(LQ)**, linear-quadratic-hinge **(LQH),** and product **(P)**. The regularization multiplier **(RM)** is a weight of penalty on model complexity. The combination of settings that produced models with the lowest value of the Akaike information criterion **(AICc)** was selected as the best model. The full **AUC** is the area under the curve for models using the full occurrences dataset (as opposed to the mean AUC, which is based on training datasets).

| CLIMATE  MODELMODEL | RMRM | FC | AUC |  | CLIMATE+  SOIL+  TOPOGRAPHIC  MODEL | RM | FC | AUC |
| --- | --- | --- | --- | --- | --- | --- | --- | --- |
| 1 | 2 | L | 0,849 |  | **1** | 0.5 | L | 0,895 |
| 2 | 1.5 | L | 0,848 |  | **2** | 1 | L | 0,891 |
| 3 | 1 | L | 0,846 |  | **3** | 1.5 | L | 0,888 |
| 4 | 0.5 | L | 0,844 |  | **4** | 2 | L | 0,887 |
| 5 | 0.5 | P | 0,856 |  | **5** | 0.5 | P | 0,962 |
| 6 | 1 | P | 0,838 |  | **6** | 1 | P | 0,932 |
| 7 | 1.5 | P | 0,817 |  | **7** | 1.5 | P | 0,902 |
| 8 | 2 | P | 0,787 |  | **8** | 2 | P | 0,897 |
| 9 | 2 | Q | 0,881 |  | **9** | 0.5 | Q | 0,950 |
| 10 | 1.5 | Q | 0,881 |  | **10** | 1 | Q | 0,949 |
| 11 | 0.5 | Q | 0,881 |  | **11** | 1.5 | Q | 0,948 |
| 12 | 1 | Q | 0,881 |  | **12** | 2 | Q | 0,946 |
| 13 | 0.5 | LQ | 0,945 |  | **13** | 0.5 | LQ | 0,974 |
| 14 | 1 | LQ | 0,944 |  | **14** | 1 | LQ | 0,973 |
| 15 | 1.5 | LQ | 0,944 |  | **15** | 1.5 | LQ | 0,971 |
| 16 | 2 | LQ | 0,942 |  | **16** | 2 | LQ | 0,969 |
| 17 | 0.5 | LQH | 0,968 |  | **17** | 0.5 | LQH | 0,988 |
| 18 | 1 | LQH | 0,961 |  | **18** | 1 | LQH | 0,986 |
| 19 | 1.5 | LQH | 0,960 |  | **19** | 1.5 | LQH | 0,983 |
| 20 | 2 | LQH | 0,959 |  | **20** | 2 | LQH | 0,982 |
| 21 | 0.5 | LQHP | 0,975 |  | **21** | 0.5 | LQHP | 0,988 |
| 22 | 1 | LQHP | 0,969 |  | **22** | 1 | LQHP | 0,986 |
| 23 | 1.5 | LQHP | 0,967 |  | **23** | 1.5 | LQHP | 0,985 |
| 24 | 2 | LQHP | 0,966 |  | **24** | 2 | LQHP | 0,983 |

**Appendix S5**: Spatial distribution models for *Butia yatay* that show the suitable areas under present and past climatic conditions using the MaxEnt and Random Forest algorithms and their overlap: Present (1979–2013), Late Holocene (LH; 4.2–0.3 ka), Mid-Holocene (MH; 8.326–4.2 ka), Early Holocene (EH; 11.7–8.326 ka), Heinrich Stadial 1 (HS; 17.0–14.7 ka), Bølling-Allerød (BA; 14.7–12.9 ka), Younger Dryas Stadial (YDS; 12.9–11.7 ka), Last Glacial Maximum (LGM; ca. 21 ka), Last Interglacial (LIG; ca. 130 ka), and Marine Isotope Stage (MIS 19; ca. 787 ka).


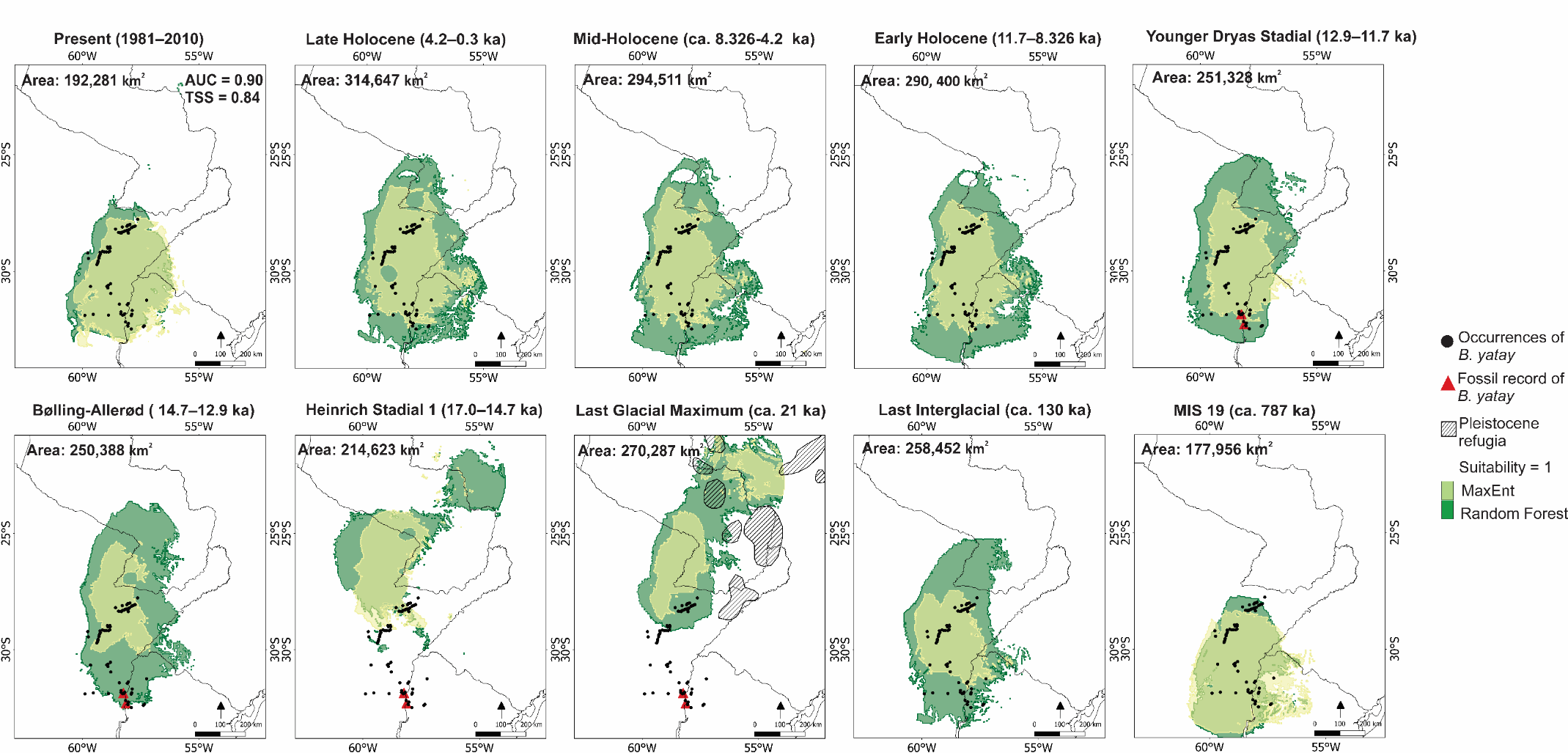


**Appendix S6**: Multivariate environmental similarity surface (MESS) analysis for paleoclimate models. MESS analysis measures climate similarity to training range when projecting a model. Negative values indicate a low similarity and therefore high climate novelty, whereas positive values indicate a high similarity and therefore low novelty.


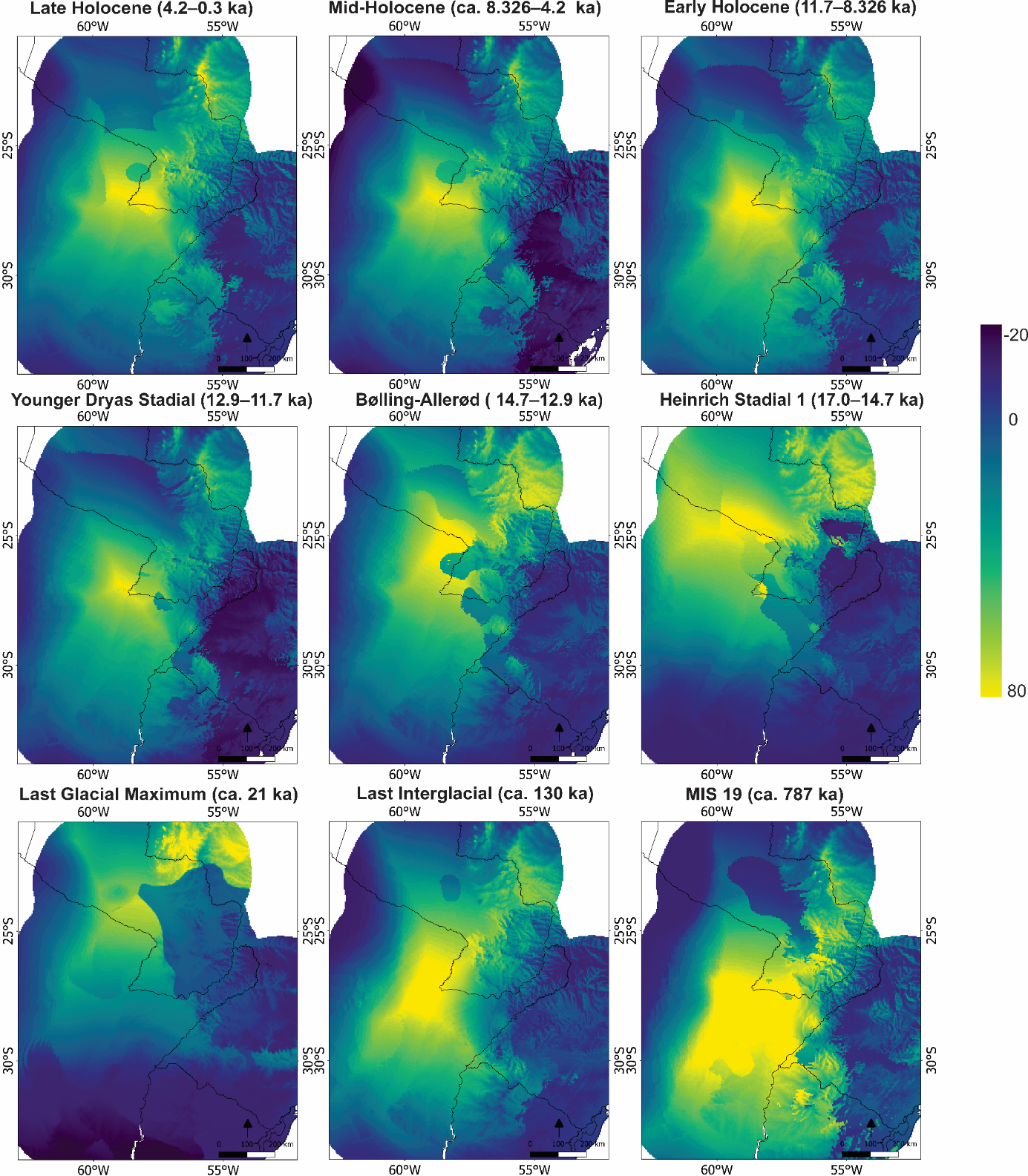

Supplement: Supplementary file 1 — Appendix S1 [file ECE3-13-e10749-s001.docx]
